# Time-Course Analysis and Transcriptomic Identification of a Group III ERF *CmTINY2* Involved in Waterlogging Tolerance in *Chrysanthemums* × *morifolium* Ramat.

**DOI:** 10.3390/ijms25158417

**Published:** 2024-08-01

**Authors:** Xueting Gu, Xinyi Liu, Haodong Sha, Xuejie Du, Han Zhang, Yuexiao Miao, Weiliang Chen, Bizeng Mao

**Affiliations:** 1 Institute of Biotechnology, College of Agriculture & Biotechnology, Zhejiang University, Hangzhou 310058, China22216251@zju.edu.cn (H.Z.);; 2 Ministry of Agriculture Key Laboratory of Molecular Biology of Crop Pathogens and Insects, Zhejiang University, Hangzhou 310058, China; 3 Zhejiang Tongxiang Hangbaiju Technology Academy, Zhejiang University, Hangzhou 310058, China; txshmzh@163.com

**Keywords:** chrysanthemum, waterlogging tolerance, time-course transcriptome, ethylene-responsive factor, CmTINY2

## Abstract

‘Hangju’ is a variety of *Chrysanthemum* × *morifolium* Ramat. with both edible and medicinal value, cultivated as a traditional Chinese medicine for four centuries. The cultivation of ‘Hangju’ is currently at risk due to waterlogging, yet there is a lack of comprehensive understanding regarding its response to waterlogging stress. This study compared the waterlogging-tolerant ‘Hangju’ variety Enhanced Waterlogging Tolerance (EWT) with the waterlogging-sensitive variety CK (‘zaoxiaoyangju’). EWT exhibited a more developed aeration tissue structure and demonstrated rapid growth regarding the adventitious roots following waterlogging. The time-course transcriptome analysis indicated that EWT could swiftly adjust the expression of the genes involved in the energy metabolism signaling pathways to acclimate to the waterlogged environment. Through WGCNA analysis, we identified *Integrase-Type DNA-Binding Protein* (*CmTINY2*) as a key factor in regulating the waterlogging tolerance in EWT. CmTINY2, a transcription factor belonging to the ethylene-responsive factor (ERF) subfamily III, operated within the nucleus and activated downstream gene expression. Its role in enhancing the waterlogging tolerance might be linked to the control of the stomatal aperture via the *Ethylene-Responsive Element* (*ERE*) gene. In summary, our research elucidated that the waterlogging tolerance displayed by EWT is a result of a combination of the morphological structure and molecular regulatory mechanisms. Furthermore, the study of the functions of *CmTINY2* from ERF subfamily III also broadened our knowledge of the role of the *ERF* genes in the waterlogging signaling pathways.

## 1. Introduction

The plant known as ‘Hangju’ is a variety of *Chrysanthemum* × *morifolium* Ramat. with a history of cultivation in traditional Chinese medicine spanning four centuries, ‘Hangju’ is recognized as one of the “Four Famous Chrysanthemums” alongside ‘Chuju’, ‘Haoju’, and ‘Gongju’. According to the Chinese Pharmacopoeia, these varieties are esteemed for their therapeutic properties, such as dispelling cold, clearing heat detoxification, calming the liver, and improving vision [1,2,3,4,5]. With the growing desire for life quality and health, ‘Hangju’ has also become an increasingly popular beverage option, offering a tasty alternative that provides health benefits as well. Hence, it is regarded as a “One Root of Medicine and Food” dietary herbal medicine with significant economic value [6]. As a plant with shallow roots, ‘Hangju’ thrives in sandy soils but is vulnerable to waterlogging stress. Its primary cultivation region in Tongxiang, Zhejiang Province, faces recurrent threats from typhoons and floods, which can lead to reduced yields, compromised quality, and, in severe cases, complete crop loss. Despite the fact that ‘Hangju’ is identified as the most waterlogging-tolerant variety among the 54 germplasm resources of tea chrysanthemums [7], waterlogging remains a significant challenge in its cultivation.

The rise in global temperatures has led to more frequent severe weather events, resulting in various natural disasters and impacting agricultural production. Notably, flooding incidents constituted the largest proportion, at 44%, of all the natural disasters recorded from 2000 to 2019 [8]. Around 27% of the global arable land is impacted by flooding annually, with the frequency of floods showing a consistent upward trend over the last six decades [9]. When waterlogging occurs, the primary consequence on plants is the hindrance of gas exchange, which results in the inhibition of aerobic respiration, thereby limiting energy metabolism and impeding plant growth. This condition also triggers anaerobic respiration, leading to the accumulation of harmful substances like lactic acid and ethanol, along with an elevation in the reactive oxygen species (ROS) levels [10]. Moreover, waterlogging disrupts the equilibrium of phytohormones such as ethylene, impacting the overall metabolic status, ultimately culminating in plant senescence or death [11,12]. To survive in such adverse conditions, plants like chrysanthemums adjust their morphological structure, energy metabolism, hormone production, and signaling mechanisms to adapt. For instance, a resilient chrysanthemum variety (‘53-4’) developed more robust adventitious roots and maintained higher levels of antioxidant enzymes to protect against oxidative damage [13]. Another waterlogging-tolerant species, *Dendranthema zawadskii,* also formed adventitious roots and developed aerenchyma to survive in low-oxygen conditions. Moreover, *D. zawadskii* showed an increase in the pyruvate decarboxylase (PDC) catalyzing and alcohol dehydrogenase (ADH) activity during waterlogging, indicating that alcohol fermentation is crucial for its ability to withstand waterlogging [14,15].

Physiological alterations in metabolism often result from the regulation of pivotal genes. In chrysanthemums, specific regulatory genes associated with waterlogging tolerance have been identified and studied, such as *SALT OVERLY SENSITIVE1* (*CmSOS1*), *MULTIPROTEIN BRIDGING FACTOR 1c* (*CmMBF1c*), *ETHYLENE-RESPONSIVE FACTORS4/5* (*CmERF4/5*), *RELATED TO AP2.3* (*CmRAP2.3*), and *HYPOXIA-RESPONSIVE ERF2* (*CmHRE2*) [15,16,17,18]. CmMBF1c directly interacts with CmHRE2, while CmSOS1 interacts with RADICAL-INDUCED CELL DEATH1 (CmRCD1), thereby enhancing the antioxidant enzyme activities, reducing the ROS levels, and improving the waterlogging tolerance [17,18]. CmERF5 binds to the promoter region of *CmRAP2.3* to stimulate its expression and alleviate the waterlogging-induced stress [16]. CmERF4 functions as a transcriptional repressor that adversely impacts the waterlogging tolerance in chrysanthemums by influencing the ROS and energy metabolism pathways [15]. Notably, *CmHRE2*, *CmRAP2.3*, and *CmERF4/5* are all members of the ERF supergene family, highlighting the significant role of ERFs in regulating the waterlogging tolerance in chrysanthemums.

The AP2/ERF transcription factor family in plants, characterized by a conserved APETALA2 domain, plays a crucial role in various stress responses, with subfamily VII being particularly linked to the waterlogging response [19]. In *Arabidopsis thaliana*, VII ERF proteins like RAP2.12 and HRE are selectively stabilized under hypoxic conditions to regulate hypoxia-responsive genes [20,21]. Meanwhile, the VII ERF genes in rice, such as *SUBMERGENCE1A* (*Sub1A*) and *SNORKEL1/2* (*Skl1/2*), modulate the internode elongation to adapt to flooding stress [22,23]. Apart from subclade VII, other ERF transcription factors may also play a role in regulating the waterlogging tolerance. For instance, *MaRAP2-4* from *Mentha arvensis* (subclade I) enhances the waterlogging tolerance by activating the expression of the *SUGAR WILL EVENTUALLY BE EXPORTED TRANSPORTER* (*SWEET10*) [24]. In addition, the expressions of the ERF subfamily III and VI genes in *Sesamum indicum* L. significantly increase after the waterlogging treatment [25]. However, further research is needed to elucidate their functions.

The deteriorating climate conditions and frequent occurrences of flooding pose a continuous threat to chrysanthemum production, but the research on the waterlogging tolerance of chrysanthemums remains limited. To date, the existing waterlogging tolerance genes in chrysanthemums are all derived from ornamental varieties [15,16,17,18,26], with few studies conducted on tea chrysanthemums like ‘Hangju’. To address this deficiency, we conducted a study on a novel cultivar of ‘Hangju’ called Enhanced Waterlogging Tolerance (EWT), which showed robust resistance to waterlogging through seedling inundation trials and field yield assessments. Additionally, we compared the changes in the gene expression after waterlogging between EWT and a waterlogging-sensitive variety (‘zaoxiaoyangju’) through time-course transcriptome analysis. The study aimed to elucidate the mechanisms underlying the waterlogging tolerance in ‘Hangju’ by examining the morphological characteristics and metabolic alterations, and it identified the transcription factor *CmTINY2* as a key player in waterlogging tolerance.

## 2. Results

### 2.1. EWT Showed Greater Tolerance to Waterlogging Than Conventional Variety

**Figure 1:**
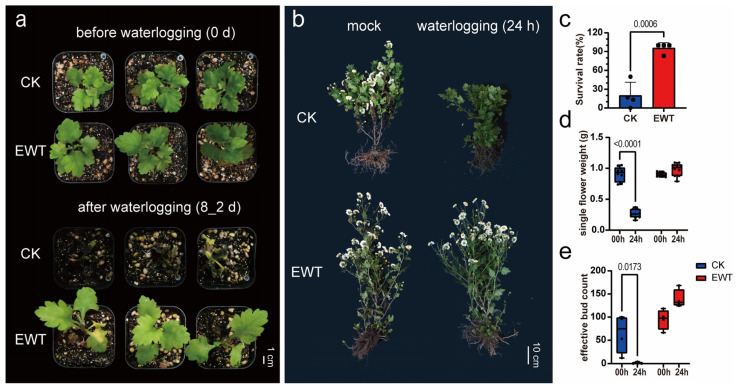
EWT shows strong waterlogging tolerance. (**a**) The seedling-stage phenotype of two ‘Hangju’ cultivars, EWT and CK, before (**top panel**) and after (**bottom panel**) waterlogging treatment. Scale bars, 1 cm. (**b**) The harvest-stage phenotypes of EWT and CK with and without 24 h waterlogging treatment in the field. Scale bars, 10 cm. (**c**) The survival rate of chrysanthemum seedlings treated with waterlogging. The average values (±s.d., *n* = 4, biologically independent samples) are shown. (**d**,**e**) The weight of single flower (**d**) and the number of effective buds (**e**) of EWT and CK with or without 24 h waterlogging treatment in the field. Data are displayed as box and whisker plots with individual data points (*n* > 3, biologically independent samples). The error bars represent maximum and minimum values. Center line, median; box limits, 25th and 75th percentiles. Significant differences were determined by Student’s *t*-test (**c**) or two-way ANOVA (**d**,**e**) with Tukey’s HSD post hoc analysis. Exact *p*-values are provided above the bars. CK refers to ‘zaoxiaoyangju’; EWT refers to a new chrysanthemum cultivar called ‘Enhanced Waterlogging Tolerance’.

To test the tolerance of chrysanthemums to complete submergence, a waterlogging treatment was performed on two ‘Hangju’ cultivars, EWT and CK (referred to as ‘zaoxiaoyangju’, the conventional cultivar planted in Tongxiang). The results showed that, following the waterlogging treatment, EWT maintained some leaves at the top to sustain growth, achieving a survival rate of 95.83%. In contrast, CK exhibited leaf crumpling and rotting, leading to a gradual decline in health, with a survival rate of only 20% (Figure 1a,c). These findings demonstrated that the waterlogging tolerance of the EWT seedlings was significantly superior to that of the conventional cultivar.

Waterlogging stress not only results in the mortality of chrysanthemum seedlings but also hampers the chrysanthemum yield by influencing the expression of the genes related to flower bud differentiation [27,28]. To investigate this phenomenon, a field flooding experiment was conducted. Following a 24 h flooding period, CK exhibited a significant reduction in the yield, as evidenced by the decreased single flower weight (Figure 1d) and effective bud number (Figure 1e). In contrast, the EWT group did not display any morphological changes or yield loss (Figure 1b), indicating its better tolerance to waterlogging stress compared to CK. Additionally, the comprehensive yield assessments revealed that, even though EWT exhibited a smaller petal number and flower diameter, its yield exceeded that of CK by 88% due to a higher number of effective flowers (Supplementary Figure S1), which might reflect EWT’s enhanced adaptability to the environmental conditions. In summary, EWT demonstrated an elevated survival rate following the waterlogging stress at the seedling stage and mitigated the yield losses following the flooding at the adult stage, highlighting its exceptional waterlogging tolerance characteristics.

### 2.2. EWT Exhibited a Well-Developed Aeration Tissue Structure

**Figure 2:**
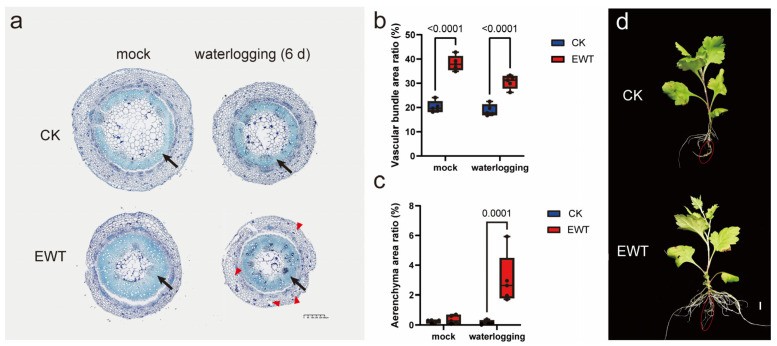
EWT has a more developed aeration tissue structure. (**a**) Transverse sections of stem bases of EWT and CK before and after waterlogging. Scale bars, 400 μm. (**b**) The percentage area of vascular bundles before and after flooding (black arrows in (**a**)). (**c**) The percentage area of aeration tissues before and after flooding (red arrows in (**a**)). Data are displayed as box and whisker plots with individual data points (*n* = 5, biologically independent samples). Exact *p*-values are indicated above the bars. (**d**) Four-week waterlogging-induced aerial roots in ‘Hangju’ (old roots are shown as red circle and the rest are new aerial roots). Scale bars, 1 cm. CK refers to ‘zaoxiaoyangju’; EWT refers to a new chrysanthemum cultivar called ‘Enhanced Waterlogging Tolerance’.

To cope with hypoxic conditions and enhance the oxygen diffusion to submerged organs, plants undergo various morphological adaptations, such as aerenchyma formation and adventitious root development [12]. The analysis of the stem base morphology through the transverse sections of the two materials before and after the waterlogging revealed a notably greater area of xylem in the EWT stems compared to the CK stems (Figure 2a,b), indicating a more robust vascular system in EWT. Additionally, some gas spaces between the cells in the stem cortex were observed in the waterlogged EWT plants, potentially indicating the formation of aerenchyma-lacunae resulting from the lysogenic cleavage of the inner cells. The measurement of the proportion showed a significantly larger area of aeration tissue in EWT compared to CK after waterlogging (Figure 2a,c). This could also facilitate gas transport and alleviate the waterlogging stress.

Furthermore, after subjecting the roots of the chrysanthemum seedlings to a two-week period of waterlogging treatment, a few adventitious roots emerged from the base of the stem at the waterlogging interface of EWT. The phenotypic observations after four weeks of treatment revealed that both EWT and CK produced aerial roots for respiration instead of primary roots, with the new aerial roots of EWT germinating earlier and displaying more advanced development (Figure 2d). These findings collectively indicated that EWT possessed a more robust morphological structure that was conducive to a waterlogging tolerance response.

### 2.3. Transcriptome Profiles of EWT and CK Exposed to Waterlogging Treatment

To explore the genomic differences in the responses to the waterlogging and reoxygenation between the sensitive (CK) and tolerant (EWT) cultivars, a total of 40 RNA-seq libraries were established for CK and EWT across five distinct waterlogging time points: 0 h, 3 h, 12 h, 24 h, and 24_3 h (representing 24 h of waterlogging followed by 3 h of reoxygenation recovery). Each library yielded an average of 57.08 million single reads, with a range of 51.96 to 64.38 million reads, which were subsequently aligned to the recently published chrysanthemum reference genome [29]. Following the quality control and filtering procedures, a total of 148,321 genes were identified in at least one sample. To conduct a comprehensive comparison of the transcriptomes from all 40 samples, a principal component analysis (PCA) was executed. The first two principal components (PC1 and PC2) collectively explained 33% of the total variance, with PC1 contributing 17.5% and effectively segregating the samples based on the duration of the waterlogging treatment, while PC2, accounting for 15.5%, distinctly separated the EWT samples from the CK samples (Supplementary Figure S2a). Notably, within both the EWT and CK groups, the samples from the different waterlogging durations exhibited dispersion, with four replicates within each group clustering together, indicating significant differences in the gene expression changes induced by the treatment and the suitability of the resulting data for subsequent analyses (Supplementary Figure S2a–c). Subsequently, differentially expressed genes (DEGs) were identified using the filter criteria of a *p*-value < 0.05 and a fold-change ≥ 2 at different time points and between the two materials at the same time. In the CK cultivar, the number of DEGs increased with prolonged waterlogging and decreased after the treatment removal. Conversely, in the EWT cultivar, the number of DEGs, both upregulated and downregulated, was higher at 12 h compared to 24 h, suggesting a potentially faster response to the waterlogging stress (Supplementary Figure S2d–f).

**Figure 3:**
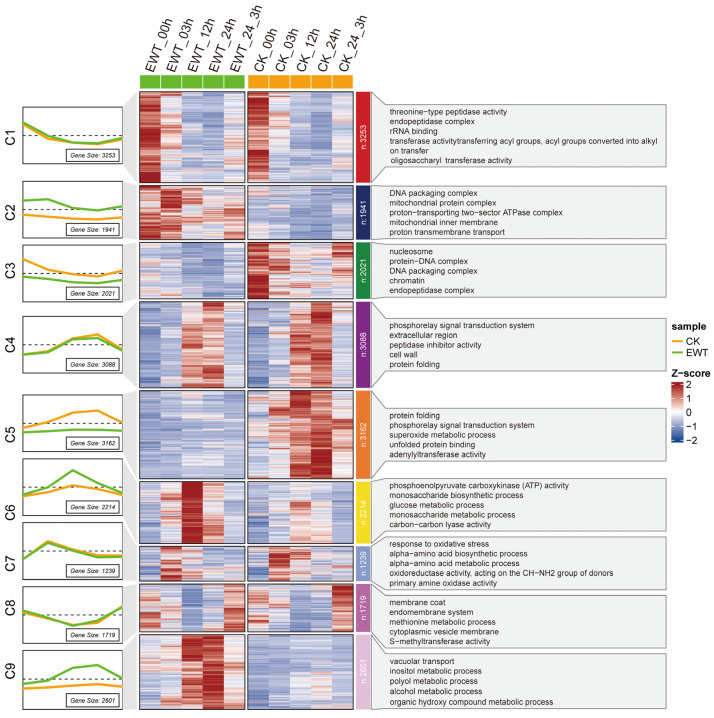
Clustering analysis of the transcriptomes of EWT and CK after waterlogging treatment. Expression pattern and functional enrichment analysis of genes in different clusters using ClusterGVis R package. The line chart (left panel) and the heatmap (middle panel) of gene expression in 9 clusters; green represents EWT root tissues, while orange represents CK root tissues. The number of genes in each cluster is shown in the lower right corner of the line chart. The top 5 functional enrichment results with the most significant differences in each cluster are listed (right panel). CK refers to ‘zaoxiaoyangju’; EWT refers to a new chrysanthemum cultivar called ‘Enhanced Waterlogging Tolerance’.

In order to characterize the temporal attributes of the entire transcriptome dataset and categorize genes with similar expression patterns, a mfuzz clustering analysis was conducted on 21,238 DEGs showing the most significant differences across 40 samples. The DEGs were categorized into nine clusters, and the top five enriched Gene Ontology (GO) pathways within each cluster were also depicted in the accompanying figure (Figure 3). Among the nine clusters, we focused on the genes that were upregulated in response to the waterlogging and showed increased expression in EWT compared to CK. These genes were considered more likely to act as positive regulators in conferring waterlogging tolerance. Notably, cluster 6 (C6) exhibited a pattern in which the gene expression levels were induced and peaked at 12 h after the treatment. The subsequent GO functional enrichment analysis revealed that the genes within the C6 group were predominantly associated with the sugar metabolism pathway, such as the “phosphoenolpyruvate carboxykinase (ATP) activity”, “monosaccharide biosynthetic process”, “glucose metabolic process”, and “monosaccharide metabolic process”. This suggests that EWT could promptly activate the energy metabolism pathway and accumulate additional energy resources to combat the stress during the initial phase (12 h) of waterlogging. On the other hand, cluster 9 (C9) exhibited a continuous increase in the gene expression levels in EWT, while only marginal changes were observed in CK. The functional enrichment analysis revealed that the genes in the C9 group were mainly associated with the alcohol metabolism pathway, such as the “inositol metabolic process”, “polyol metabolic process”, “alcohol metabolic process”, and “organic hydroxy compound metabolic process”, signifying that EWT could induce the expression of the genes involved in alcohol metabolism, which could generate energy and eliminate the toxic hydroxyls generated during the anaerobic respiration in the later stages (24 h) of waterlogging. In conclusion, the results of the functional enrichment analysis following gene clustering demonstrated that EWT could accelerate and enhance the upregulation of the genes in the energy metabolism pathways following waterlogging, thereby orchestrating waterlogging tolerance responses through a multifaceted approach (Figure 3). 

### 2.4. Mining the Waterlogging Tolerance Regulator Using WGCNA Analysis

**Figure 4:**
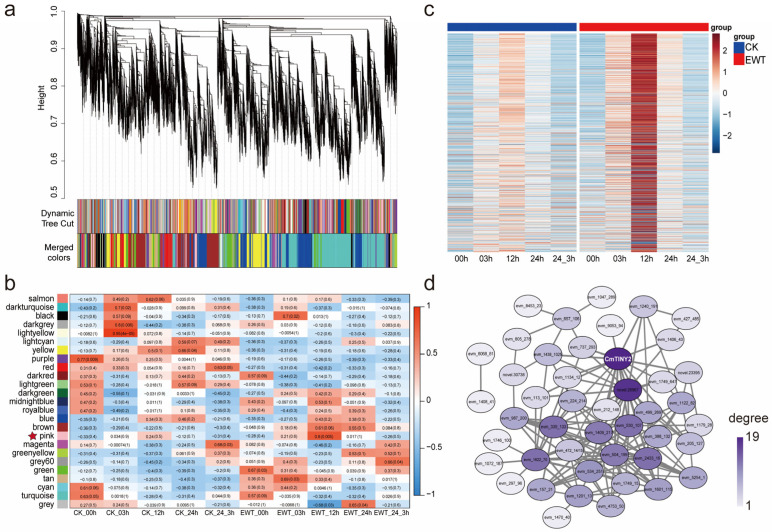
WGCNA for DEGs identified in ‘Hangju’ under waterlogging treatment. (**a**) Identification of co-expression gene modules using a hierarchical clustering tree. The 25 identified modules are indicated by different colors. (**b**) Heatmap showing the correlation between gene modules and sample groups. In each cell, the left number represents the correlation and the right number in parentheses represents the significance. The ‘pink’ module of interest is marked by asterisks. (**c**) Heatmap of the expression pattern of genes in the ‘pink’ modules. (**d**) Topological illustration of the ‘pink’ co-expression module. Line thickness indicates the strength of the connection. The darker the color, the more closely related it is to other genes and the more likely it is to be a hub gene.

To explore the key regulatory genes of EWT involved in the response to waterlogging stress, we performed a weighted gene co-expression network analysis (WGCNA) on the 36,798 DEGs and divided them into 25 modules (Figure 4a,b). The DEGs within the same module exhibited similar expression patterns, indicating tight co-regulation. The ‘pink’ module, comprising 707 DEGs with an r2 value of 0.8 and a *p*-value of 0.005, was subjected to further examination. Notably, the gene expression within the pink module showed an initial increase during the first 12 h of waterlogging, followed by a gradual decline in both experimental materials (Figure 4c). The elevation in the expression levels was more pronounced in EWT compared to CK, consistent with cluster 6 in Figure 3. The genes co-expressed in this module were significantly enriched in the small-molecule catabolic processes, such as the “alpha-amino acid catabolic process” and the “oligosaccharide metabolic process” (Supplementary Figure S3). Subsequently, the genes from the ‘pink’ module were selected for edge and node calculations, which were used for gene network visualization. This analysis led to the identification of a gene labeled as CmTINY2, represented by the dark purple color (Figure 4d).

**Figure 5:**
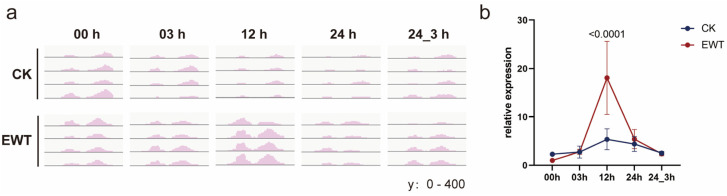
*CmTINY2* is induced during waterlogging treatment. (**a**) Genome browser view of *CmTINY2* mRNAs in the roots of CK and EWT. (**b**) qPCR analysis of the relative expression of *CmTINY2* in two materials during waterlogging. *CmActin* was used as an internal control. The average values (±s.d., *n* = 4, biologically independent samples) are shown. Significant differences were determined by two-way ANOVA with Tukey’s HSD post hoc analysis. Exact *p*-values are shown above the bars. CK refers to ‘zaoxiaoyangju’; EWT refers to a new chrysanthemum cultivar called ‘Enhanced Waterlogging Tolerance’.

The transcriptome sequencing data revealed a significant upregulation of the *CmTINY2* expression in EWT after 12 h of waterlogging, while its expression remained unaffected in CK (Figure 5a). This disparity was further validated through qPCR experiments (Figure 5b and Supplementary Figure S4). Based on these findings, *CmTINY2* was identified as a potential candidate gene induced by waterlogging stress from the transcriptome data, suggesting its potential role in waterlogging tolerance. The subsequent analysis aimed to elucidate the functional mechanism of this gene.

### 2.5. CmTINY2 Is a Transcriptional Activator of DREB Subfamily

**Figure 6:**
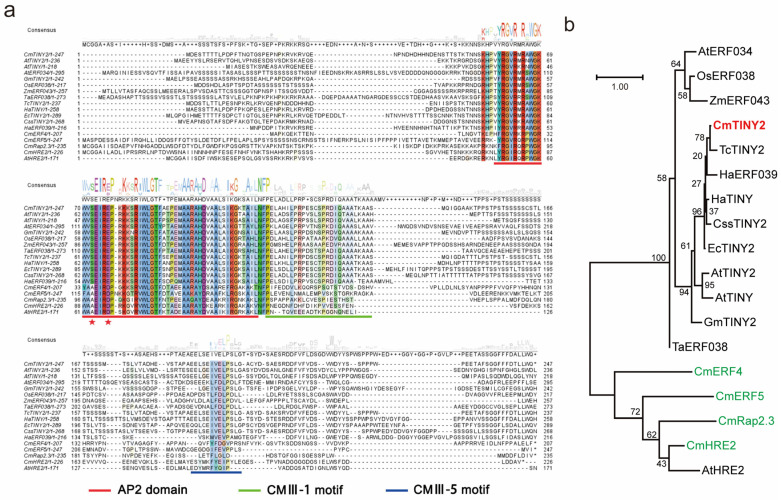
Sequence alignments and phylogenetic analysis of CmTINY2. (**a**) Polypeptide sequence alignment of CmTINY2 and its orthologous and paralogous ERF proteins. The AP2 domain is marked with a red line. The 14th (V or A) and 19th (E or D) amino acids in the AP2 domain are marked by asterisks. CMIII-1 and CMIII-5 are marked by green and blue lines, respectively. (**b**) Phylogenetic tree of CmTINY2 (marked in red) and other ERF proteins of *Arabidopsis thaliana* [AtERF034 (AT2G44940.1), AtTINY2 (AT5G11590.1), AtTINY (AT5G25810.1), and AtHRE2 (AT2G47520.1)], *Oryza sativa* [OsERF038 (XP_015634439.1)], *Zea mays* [ZmERF043 (XP_008646011.1)], *Tanacetum cinerariifolium* [TcTINY2 (GFA17976.1)], *Helianthus annuus* [HaTINY (XP_021990459.1)], *Cynara cardunculus var. scolymus* [CssTINY2 (XP_024980282.1)], *Erigeron canadensis* [EcTINY2 (XP_043624060.1)], *Glycine max* [GmTINY2 (XP_003530573.1)], *Triticum aestivum* [TaERF038 (XP_044328597.1)], and *Chrysanthemum* × *morifolium* Ramat. [CmERF4, CmERF5, CmRap2.3, and CmHRE2]. The known CmERFs associated with waterlogging tolerance are marked in green. The scale bar (1.00) indicates branch length.

We cloned CmTINY2 from cDNA isolated from the roots of the chrysanthemum cultivar ‘EWT’. The full-length ORF of CmTINY2 was 741 bp and encoded 246 amino acids. The analysis of the protein sequence indicated the presence of a conserved AP2 domain and two CMIII motifs, suggesting that CmTINY2 belonged to the ERF-IIIs group (Figure 6a) [19]. Notably, the 14th and 19th amino acids within the AP2 domain of CmTINY2 were valine (V) and glutamic acid (E), respectively, indicating its membership in the DEHYDRATION-RESPONSIVE ELEMENT-BINDING PROTEINS (DREB) subfamily [30]. This set it apart from the known CmERFs associated with waterlogging tolerance (Figure 6a). The phylogenetic assessment also demonstrated that CmTINY2 shared a cluster with *Tanacetum cinerariifolium* TcTINY2 and exhibited distant relations with other reported ERF genes in chrysanthemums (Figure 6b). Therefore, we speculated that the study of CmTINY2 would help to refine the network of ERF transcription factors that regulate the waterlogging tolerance in chrysanthemums.

**Figure 7:**
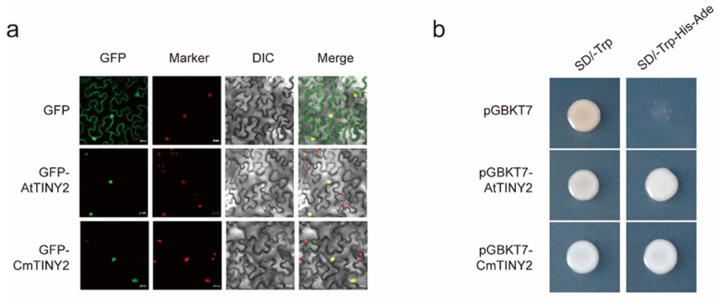
Subcellular localization and transactivation analysis of CmTINY2. (**a**) Subcellular localization of AtTINY2 and CmTINY2 in H2B-RFP transgenic *N. benthamiana* leaves. GFP, images taken in the green fluorescence channel; marker, nuclear localization with red fluorescent protein; DIC, images taken in the bright light channel; merged, overlay plots. Scale bar, 20 μm. (**b**) Analysis of the transcriptional activity of AtTINY2 and CmTINY2 in yeast cells; pGBKT7 served as a negative control.

To clarify the subcellular localization of CmTINY2, we conducted transient expression experiments in the H2B-RFP transgenic *Nicotiana benthamiana* by infiltrating leaves with an *Agrobacterium tumefaciens* suspension carrying the 35S::GFP-CmTINY2 construct. The results showed that CmTINY2 was predominantly present in the nucleus, consistent with its homolog AtTINY2 (Figure 7a). In addition, we fused CmTINY2 with the GAL4-binding domain (BD) and expressed the recombinant protein in yeast to examine its transcriptional activity. The yeast cells transformed with the BD-CmTINY2 vector exhibited viability on a SD/Ade-His-Trp- medium, indicating that CmTINY2 possessed transcriptional activity, like its homologue AtTINY2 in arabidopsis (Figure 7b) [31]. 

### 2.6. CmTINY2 Influenced Waterlogging Tolerance Probably through Stomatal Regulation

According to Nakata et al., AtTINY2 has the capacity to enhance the production of the primary cell walls and make the plant more robust [32]. In this study, an examination was conducted on the stem phenotype in ‘Hangju’. However, no notable disparity in the vessel cell wall thickness was observed between the two materials (Supplementary Figure S5). The lack of a significant difference might be attributed to the limited duration of the CmTINY2 induction through the waterlogging treatment, which was insufficient to induce morphological alterations.

**Figure 8:**
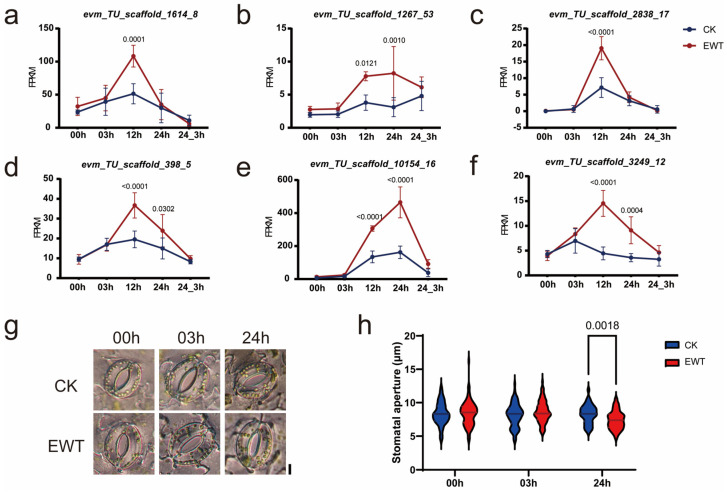
The expression of DRE and ERE genes and the stomatal aperture of ‘Hangju’ under different stages of waterlogging treatment. (**a**–**f**) Expression of DRE (**a**–**c**) and ERE (**d**–**f**) genes in two materials at the indicated time points during waterlogging. Data points show expression levels as average FPKM (fragments per kilobase of exon per million fragments mapped) in four biological replicates. The average values (±s.d., *n* = 4, biologically independent samples) are shown. (**g**) Representative images of stomata on the lower surface of leaves when EWT and CK were completely submerged for 0, 3, and 24 h. Scale bar, 10 μm. (**h**) Stomatal apertures of EWT and CK leaves (each replicate quantified 80 stomata from three individual leaves). Significant differences were determined by two-way ANOVA with Tukey’s HSD post hoc analysis. Exact *p*-values are indicated above the bars. CK refers to ‘zaoxiaoyangju’; EWT refers to a new chrysanthemum cultivar called ‘Enhanced Waterlogging Tolerance’.

Based on the protein sequence analysis, TINY2 belongs to the DREB subfamily. However, it can activate the expression of the downstream DRE and *Ethylene-Responsive Element* (*ERE*) genes simultaneously [30]. AtTINY2 binds to ERE via the 15th amino acid in the AP2 domain and binds to the DRE gene via the 14th and 19th amino acids, which are the same in AtTINY2 and CmTINY2 (Figure 6). We also found that some DRE genes in *Chrysanthemum × morifolium* Ramat. with a core sequence of A/GCCGAC in the promoter region were upregulated after 3 h of waterlogging, as well as some other ERE genes containing the AGCCGCC sequence (Figure 8a–f), and they might be directly downstream of CmTINY2. In *Arabidopsis thaliana*, the homolog of evm_TU_scaffold_398_5, one of the ERE genes has been reported to be involved in the regulation of stomatal vapor pressure difference signaling [33]. Thus, the stomatal size of both plant materials was assessed after 0, 3, and 24 h of waterlogging treatment. Remarkably, the stomatal aperture of EWT decreased significantly after 24 h of waterlogging, whereas this effect was not observed in CK (Figure 8g,h). Given the increased expression of *evm_TU_scaffold_398_5* during the 3 h to 24 h period of waterlogging, we postulated that CmTINY2 could potentially impact the regulation of the stomatal aperture by influencing the expression of *evm_TU_scaffold_398_5*, thereby modulating the waterlogging tolerance response.

## 3. Discussion

### 3.1. EWT Shows Excellent Waterlogging Tolerance

Waterlogging removes air from the soil pores, impeding the gas exchange between the soil and the atmosphere. This limitation results in decreased root respiration, suppressed root activity, and energy deficiency [34]. In our study, EWT showed higher respiratory activity after waterlogging by activating crucial genes related to the alcohol metabolism pathway and the sugar metabolism pathway. This mechanism boosted the plant’s energy supply and assisted in eliminating the toxic substances accumulated due to hypoxia. Prior research indicates a strong correlation between the capacity of plants to endure waterlogging conditions and their energy metabolism pathways [10,13]. For example, the enzyme ADH, crucial in ethanol metabolism, is notably affected by waterlogging stress in different plants, such as *Tritcum aestivum* [35], *Cerasus sachalinensis* [36], and *Chrysanthemum* × *morifolium* [13,26]. The waterlogging-sensitive plants *CmERF4*-OX also show reduced expression of *ADH* and other genes associated with energy metabolism like *PYRUVATE KINASE* (*PK*) and *HEXOKINASE-2-like* (*HK2-like*) [15]. As the specific signaling pathways involved remain unclear, future investigations will delve deeper into the pivotal genes governing the energy metabolism following waterlogging in EWT, aiming to elucidate the underlying mechanism.

As waterlogging progresses, the deteriorating primary roots lose their capacity to supply the plant with essential water and nutrients [37]. Consequently, the emergence of adventitious roots serves as an adaptive mechanism in plants to address this issue [13]. Our research findings also revealed that EWT exhibited more accelerated and more advanced aerial root growth compared to CK following the waterlogging treatment. Moreover, the microscopic analysis indicated that EWT displayed enhanced vascular bundles and more mature aerial tissues, suggesting a morphological and structural basis for the waterlogging tolerance. This observation further elucidated the heightened adaptability of EWT to inundation.

Tang et al. utilized simulated waterlogging in pots to assess the waterlogging tolerance of 54 tea chrysanthemum varieties and identified ‘Hangju’ as the most waterlogging-tolerant variety based on the average membership function values of waterlogging (MFVW) [7]. Our study corroborated these findings, demonstrating the robust waterlogging tolerance of ‘Hangju’. For instance, the seedlings subjected to 8 days of waterlogging only perished when completely submerged, while those with flooded roots were able to develop aerial roots to sustain growth, as observed in both the EWT and CK materials. Nevertheless, it is important to highlight that root respiration inhibition and the accumulation of harmful substances during waterlogging stress adversely impacted both the vegetative and reproductive growth. The field trials confirmed a notable decrease in the CK yield after 24 h of flooding despite the plant survival. High levels of soil moisture content have been observed to have a suppressive effect on both the ray florets (RFs) and disc florets (DFs) within the inflorescence morphology. Furthermore, the altered expression of the *FLAVONOL SYNTHASE* (*FLS*) gene impacted not only the floral development but also the quality of the ‘Hangju’ products [28]. Due to the increasingly severe weather patterns and frequent instances of flooding [9], there is considerable potential for the widespread adoption of waterlogging-resistant cultivar EWT, which has shown the ability to sustain high yields and ensure consistent production. On the other hand, further exploration of this cultivar may lead to the discovery of crucial waterlogging-resistant genes in chrysanthemums and the elucidation of the signaling regulatory mechanisms that enable chrysanthemums to cope with flooding events.

### 3.2. CmTINY2 Is an ERF Transcription Factor Induced by Waterlogging Treatment

Through WGCNA, the key regulatory gene *CmTINY2* associated with waterlogging tolerance was identified in EWT. The subsequent protein sequence analysis revealed its classification within the III subfamily of ERF transcription factors. The subgroup III genes, to which CmTINY2 belongs, have been extensively researched and are known to be involved in various abiotic stresses, such as low-temperature stress, salt stress, and drought stress [19,38,39,40]. For instance, the subgroup III gene *ERF5* in tomato is upregulated in response to abiotic stresses such as flooding. The transgenic lines overexpressing this gene exhibit enhanced resistance to abiotic stresses [41]. The investigations on *Medicago sativa* L. and *Sesamum indicum* L. have also indicated that the genes within the III subfamily are likely associated with the functions related to abiotic stresses [25,42].

The signaling pathway of the subfamily VII ERFs involved in enhancing the waterlogging tolerance in plants has been extensively studied. The genetic roles of the VII ERFs in chrysanthemums, such as CmHRE2 and CmRAP2.3, have been confirmed in previous research [16,18]. Recent investigations have also identified the participation of CmERF4 from the subfamily VIII ERFs and CmERF5 from the subfamily IX ERFs in waterlogging tolerance mechanisms [15,16]. Our experiments examined the gene expression levels through transcriptome analysis and qPCR validation. The results revealed that *CmHRE2*, *CmRAP2.3*, and *CmERF4* were upregulated under waterlogging conditions, with higher expression in EWT compared to CK. It has been reported that *CmHRE2* and *CmRAP2.3* play positive roles in enhancing waterlogging tolerance, potentially contributing to the tolerance phenotype of EWT. While CmERF4 acts as a negative regulator, its elevated expression may suggest a negative feedback mechanism aimed at reaching equilibrium, which requires additional experimental validation.

### 3.3. CmTINY2 Might Be Involved in the Regulation of Stomatal Movement in EWT

To explore the role of *CmTINY2* in waterlogging tolerance, we cloned the gene with its homologous *AtTINY2* in *Arabidopsis thaliana*. The experiments showed that both proteins were localized in the nucleus and exhibited transcriptional activating activity. Previous research has demonstrated that the overexpression of AtTINY2-VP16 can induce cell wall thickening and modify lignin composition and deposition [32]. In this study, we assessed the cell wall thickness of both materials before and after the waterlogging treatment; however, no discernible differences were observed.

Stomata play a crucial role in plant physiology as they are vital for processes such as photosynthesis and respiration. In chrysanthemums, it has been observed that waterlogging induces stomatal closure [43], and waterlogging-tolerant varieties always exhibit earlier stomatal closure compared to waterlogging-sensitive varieties [44]. It has also been reported that AtTINY positively regulates the drought response by inducing the expression of drought-responsive genes and promoting abscisic acid (ABA)-regulated stomatal closure [40]. Our findings indicated that, after 24 h of waterlogging, EWT demonstrated greater efficacy in inducing stomatal closure compared to CK. This might be associated with the upregulation of a potential downstream ERE gene *evm_TU_scaffold_398_5*. According to protein BLAST, the homologous gene of *evm_TU_scaffold_398_5* in *Arabidopsis thaliana* is *RAF6*, a mitogen-activated protein (MAP) kinase. Research has indicated that *RAF6* plays a role in the complex mechanism of stomatal closure mediated by the leaf-to-air vapor pressure difference [33]. Nevertheless, the direct regulatory relationship between the three entities must be validated with further experimental data.

## 4. Materials and Methods

### 4.1. Plant Materials

A common cultivar of *Chrysanthemum* × *morifolium* cv. ‘Hangju’ named ‘zaoxiaoyangju’ was used as the waterlogging-sensitive control (CK) in this study. Both EWT and CK were stored and propagated from cuttings in the field of Tongxiang, YuanYuan Edible Flower Professional Cooperative (120.46° E, 30.62° N; altitude: 5 m). The cultivation of aseptic ‘Hangju’ seedlings was described by Yan et al. [45]. The experiment was conducted in a greenhouse at 28 °C with a 12 h/12 h photoperiod. *Nicotiana benthamiana* for transient expression was grown at 22 °C with a 12 h/12 h photoperiod.

### 4.2. Waterlogging Treatment of Chrysanthemums

For the seedling waterlogging treatment, 8-week-old seedlings of EWT and CK were subjected to waterlogging stress by submerging them in water up to 2 cm above the upper leaves (most of experiments in our study) or soil surface (only for Figure 2d). After 8 days of continuous waterlogging, the water was drained and the plants were kept under normal growth conditions. Two days later, the plants were photographed to record the phenotype and the survival rate was counted. Experiments were independently repeated four times with more than four individuals of each material.

For the field waterlogging treatment, 4-month-old plants of EWT and CK were subjected to field flooding by submerging them in water up to 5 cm above the soil surface for 24 h in September, and the yield of each field was measured at the full flowering stage on 10 November from more than 5 individual plants.

### 4.3. RNA Extraction, RNA-Seq Library Construction, and Sequencing

Root samples of EWT and CK seedlings were collected immediately at 0, 3, 12, and 24 h after waterlogging and 24 h after waterlogging with 3 h of reoxygenation (24_3 h). Each sample contained four biological replicates. Total RNA was extracted using the Tiangen RNA Extraction Kit with the RNase-Free DNase Set (Tiangen Biotech, Beijing, China) according to the manufacturer’s instructions.

Libraries for mRNA-seq were constructed and sequenced by Novogene (Beijing, China). Briefly, RNA integrity was assessed using the RNA Nano 6000 Assay Kit of the Bioanalyzer 2100 system (Agilent Technologies, Santa Clara, CA, USA). mRNAs were isolated from total RNAs by poly(A) selection, fragmented into short fragments, and converted to cDNAs. cDNAs were ligated to adapters and the suitable fragments were selected for PCR amplification as templates. All the RNA-seq libraries were pair-end sequenced on an Illumina Novaseq platform. All raw data generated in this research have been submitted to the Gene Expression Omnibus (GEO) repository under GSE269106.

### 4.4. RNA-Seq Data Analysis

Analysis of the mRNA sequencing data was performed as reported [46]. Low-quality reads were removed, and adapters were trimmed to obtain clean reads, which were mapped to the reference genome [29], using HISAT2 2.0.5. The expression level of each gene was calculated as the FPKM value (fragments per kilobase of transcript per million mapped reads). Differentially expressed genes (DEGs) were identified using the DEseq2 1.20.0 R package with filtering criteria of *p*-value ≤ 0.05 and fold change ≥ 2. Gene Ontology (GO) and Kyoto Encyclopedia of Genes and Genomes (KEGG) enrichment analysis of the annotated DEGs was performed on the NovoMagic System (https://magic.novogene.com/ (accessed on 1 August 2023)). Expression data for all genes at all submergence time points are provided in Supplementary Data S1.

Principal component analysis was performed using FactoMineR 2.9 and factoextra R 1.0.7 packages. Mfuzz clustering was conducted to classify the identified DEG expression patterns using ClusterGVis 0.1.1 R package. Weighted gene co-expression network analysis (WGCNA) was performed on the NovoMagic System (https://magic.novogene.com/). The parameters for WGCNA were set as follows: power = 10; hierarchical clustering tree, Dynamic Hybrid Tree Cut algorithm; minimum module size, 30; and minimum height for merging modules, 0.25. Co-expression networks were visualized using Cytoscape 3.10.1 software with the weight cutoff ≥ 0.4.

### 4.5. Fluorescence Quantitative Polymerase Chain Reaction (qPCR) Analysis

Total RNA (approximately 1 μg) was converted to cDNA using ReverTra Ace qPCR RT Master Mix with the gDNA Remover kit (TOYOBO, Japan) according to the manufacturer’s instructions. The qPCR was performed using a LightCycler^®^ 96 SW 1.1 (Roche, Basel, Switzerland) with SYBR Premix Ex Taq (Takara, Shiga, Japan) following the manufacturer’s instructions. *CmActin* was used as an internal control to normalize expression levels, and the 2^−ΔΔCT^ method was used to calculate the relative expression levels with four biological replicates. Primers used are listed in Supplementary Data S2.

### 4.6. Isolation and Sequence Analysis

For the construction of *CmTINY2* and *AtTINY2* overexpression plasmids, the full-length coding sequences of *CmTINY2* and *AtTINY2* were amplified from cDNA using primer pairs CmTINY2_IN_F/R and AtTINY2_IN_F/R and cloned into binary vector PCambia 1300 with GFP tag. Primers used are listed in Supplementary Data S2. 

We used Jalview 2.11.3.2 software to perform multiple sequence alignment among the CmTINY2 protein homologs from the GenBank (https://www.ncbi.nlm.nih.gov (accessed on 1 August 2023)). A Maximum Likelihood phylogeny tree was constructed using MEGA 11 software with 1000 bootstrap replicates.

### 4.7. Subcellular Localization

p35S::GFP-CmTINY2 and p35S::GFP-AtTINY2 were separately transformed into Agrobacterium strain GV3101. Overnight cultures (12–16 h) of Agrobacterium were collected by centrifugation and resuspended in MES buffer (10 mM MgCl_2_; 10 mM MES; 100 μM acetosyringone; pH 5.6). The concentration of the bacterial suspension was adjusted to OD_600_ of 0.8 and incubated at 28–30 °C for 3 h before infiltration. Leaves of 4-week-old transgenic *N. benthamiana* 35S::H2B-RFP were syringe-infiltrated with the suspensions. Confocal microscopy with Zeiss LSM 880 (Jena, Germany) was performed to observe GFP activity between 36 and 48 h. Primers used are listed in Supplementary Data S2.

### 4.8. Transcriptional Activity Analysis

The CmTINY2 and AtTINY2 full-length ORFs were inserted into the pGBKT7 vector. The plasmids including the negative control (pGBKT7 empty vector) were used to transform into AH109 yeast cells in a synthetic defined medium without tryptophan (SD/-Trp). After 3 days at 30 °C, the yeast cells were cultured on the synthetic defined medium lacking tryptophan, histidine, and adenine (SD/-Trp-His-Ade). The growth of the yeast cells in the medium was observed to determine the transcriptional activity of the plasmids. Primers used are listed in Supplementary Data S2.

### 4.9. Microscopic Observation

The stem bases (within 0.5 cm above the junction of the root and stem) of 8-week-old chrysanthemum materials before and after 6 days of waterlogging were sampled and placed in FAA (4% paraformaldehyde, 15% acetic acid, and 50% ethanol) for 24 h and then sent to Hangzhou Haoke Biotechnology Company (Hangzhou, China) for paraffin section staining and imaging. The steps were as follows. A dehydrator (Hubei Beno Medical Technology Co., Ltd., Daye, China), a biological tissue automatic embedding machine (Hubei Beno Medical Technology Co., Ltd., Daye, China), and a rotary slicer HistoCoreBIOCUT (Leica Microsystems Shanghai Co., Ltd., Shanghai, China) were utilized for the sequential processes of dehydration, embedding, and sectioning to obtain paraffin sections. The sections were immersed in xylene for 20 min, another xylene for 20 min, 100% ethanol for 5 min, another 100% ethanol for 5 min, and finally in 75% ethanol for 5 min before being washed with water. Subsequently, the sections were stained with toluidine blue dye for 3–6 min [47], washed again, and dried in the oven HPF-50 (Shanghai Yuejin Medical Equipment Co., Ltd., Shanghai, China). Put the sections into xylene for 5 min to clear, remove the sections from the xylene to dry slightly, and seal the sections with neutral gum. Image acquisition and analysis were performed using the microscope ECLIPSE E100 (Nikon, Tokyo, Japan). Five seedlings from each group were selected, and one sample was taken from the base of each seedling. This experiment was repeated three times independently.

To investigate the effects of waterlogging on stomatal movement, 8-week-old chrysanthemum materials were exposed to light for 3 h. Once the stomata were fully open, the plants were submerged. At each designated time point, the epidermal strips were immediately peeled from the abaxial surface of the expanded leaves and stomatal aperture images were captured using a Nikon Eclipse Ni microscope equipped with a Nikon DS-Fi2 digital camera (Japan). For each type, we selected three leaf samples to observe, measuring a total of 80 stomatal diameters. Adobe Photoshop 2020 was utilized for measuring specific areas. This experiment was repeated three times independently.

### 4.10. Statistical Analysis

For qRT-PCR analyses, waterlogging tolerance assays, agronomic trait assessments, and physiological assays, significant differences between experimental group and the corresponding controls were analyzed using two-tailed Student’s *t*-test for pairwise comparisons or two-way ANOVA analysis with Tukey’s multiple comparison test as specified in the figure legends. Detailed information about statistical analysis values for all analyses is provided in Supplementary Data S3.

### 4.11. Accession Numbers

Sequencing data of this study are available at the National Center for Biotechnology Information (https://www.ncbi.nlm.nih.gov/bioproject/ (accessed on 23 July 2024)) with the GEO accession number GSE269106.

## Data Availability

All data generated or analyzed during this study are included in this published article and its Supplementary Files. The datasets generated and analyzed during the current study are available from the corresponding author upon reasonable request.

## Supplementary Materials

The following supporting information can be downloaded at https://www.mdpi.com/article/10.3390/ijms25158417/s1.
