# Supplementary material for: Time-Course Analysis and Transcriptomic Identification of a Group III ERF CmTINY2 Involved in Waterlogging Tolerance in Chrysanthemums × morifolium Ramat
Source: Int J Mol Sci. 2024 Aug 1;25(15):8417. doi: 10.3390/ijms25158417 (PMC11312920; doi:10.3390/ijms25158417)
Supplement: Supplementary file 1 [file ijms-25-08417-s001.zip › Supplementary Figures.pdf]

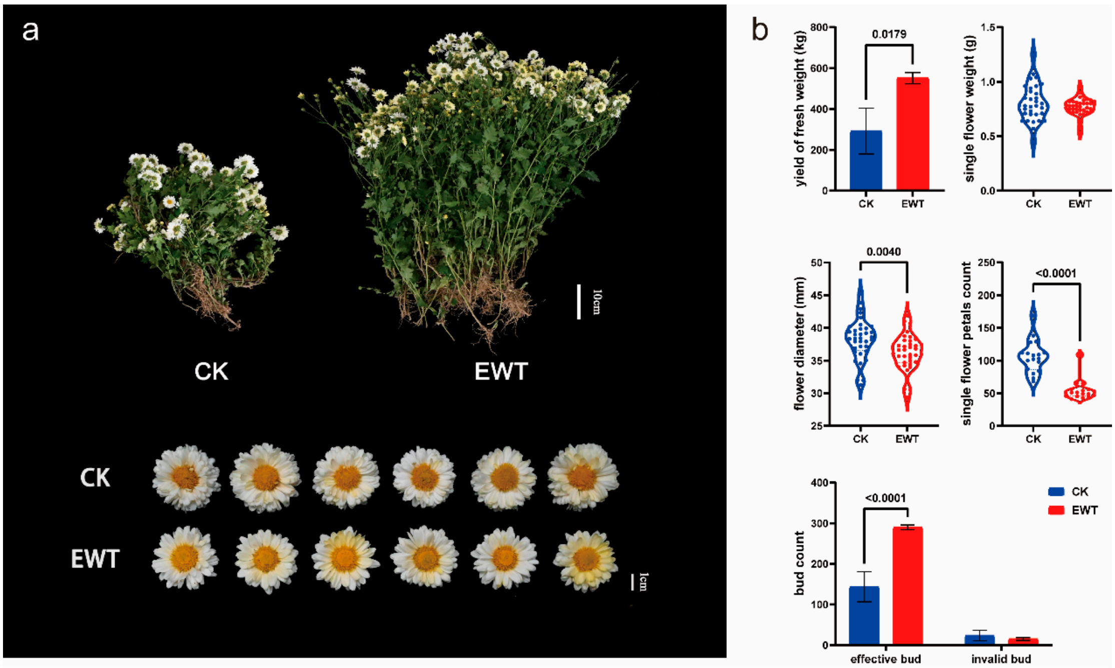

Supplementary Figure S1. Phenotypes and yield traits of EWT.

(a) Phenotype of plants and flowers of ‘hangju’ cultivar EWT and CK at harvest. (b) Yield traits such as fresh weight per mu, single flower weight, flower diameter, number of petals and number of buds of EWT and CK in the yield. The average values (±s.d., $n \geqslant 3$, biologically independent samples) are shown. Significant differences were determined by Student's t-test or two-way ANOVA with Tukey’s HSD post hoc analysis. Exact P values are indicated above the bars. CK refers to 'zaoxiaoyangju', EWT refers to a new chrysanthemum variety called 'Enhanced Waterlogging Tolerance'.

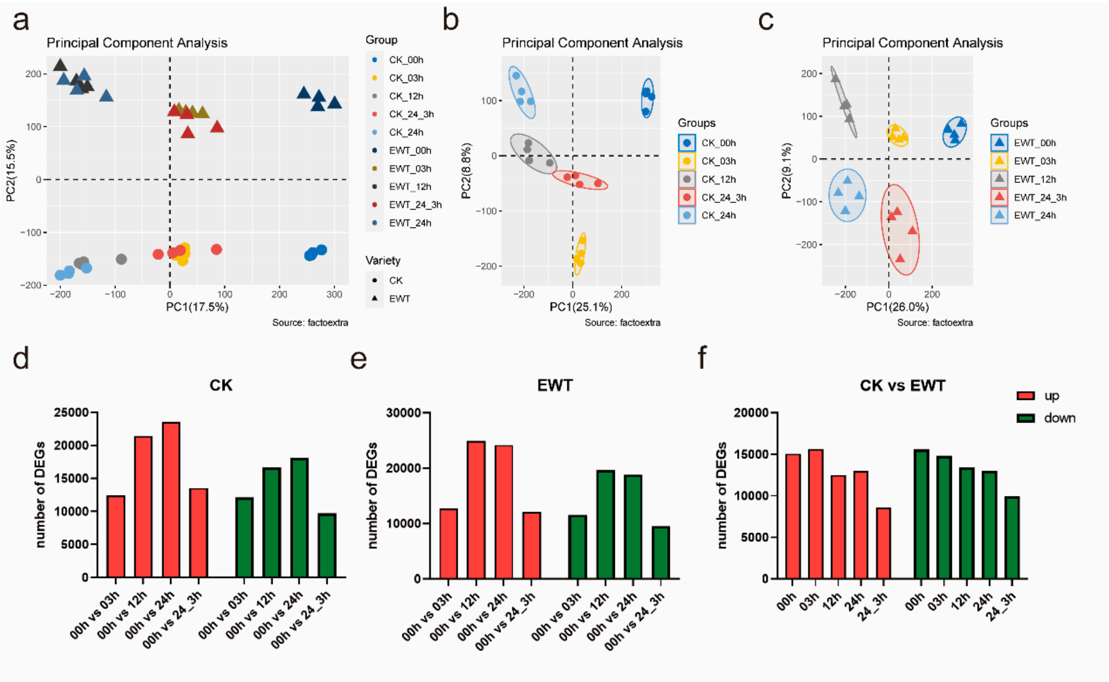

Supplementary Figure S2. The PCA analysis and DEG number of 'hangju' transcriptome after waterlogging.

(a-c) Principal component analysis results of all samples (a), CK (b), and EWT (c) based on all transcripts identified by RNA-seq. Circles represent CK materials, triangles represent EWT materials, and waterlogging duration is indicated by color. Each treatment included 4 biological replicates. PC1, principal component 1; PC2, principal component 2. The ellipse represents the 0.75 confidence level. (d-f) Number of differentially expressed genes (DEGs) (|fold change|≥ 2, p≤0.05) identified by RNA-seq during waterlogging treatment of CK (d), EWT (e) and between the two materials (f). CK refers to 'zaoxiaoyangju', EWT refers to a new chrysanthemum variety called 'Enhanced Waterlogging Tolerance'.

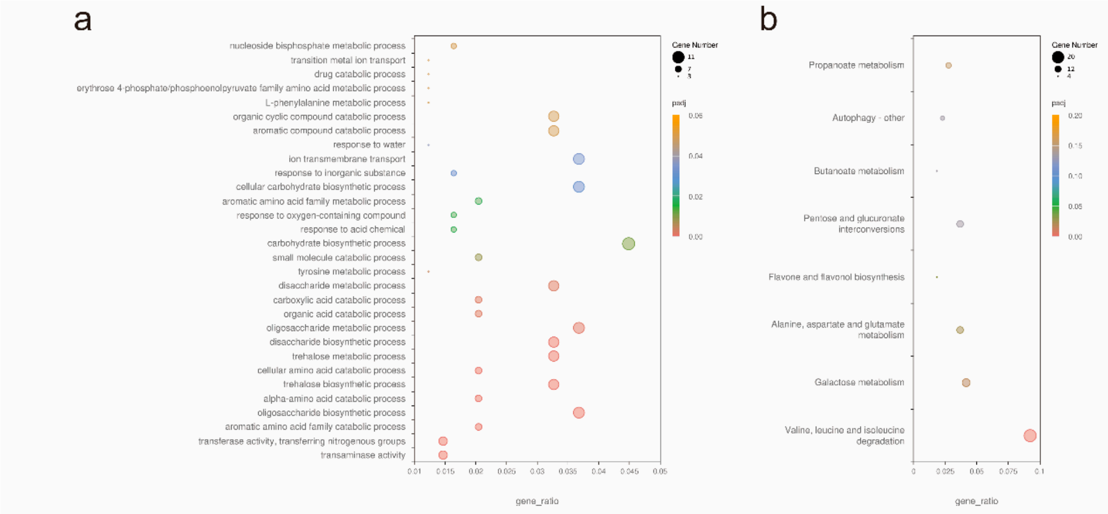

Supplementary Figure S3. GO term and KEGG enrichment analysis of genes in the ‘pink’ module.

(a) Gene Ontology (GO) term enrichment analysis. (b) Kyoto Encyclopedia of Genes and Genomes (KEGG) enrichment analysis. The x-axis represents the gene ratio, while the y-axis represents the enrichment pathway. The dot sizes represent the number of differentially enriched genes. The statistical analysis of the pathway enrichment was performed using a Fisher’s exact test.

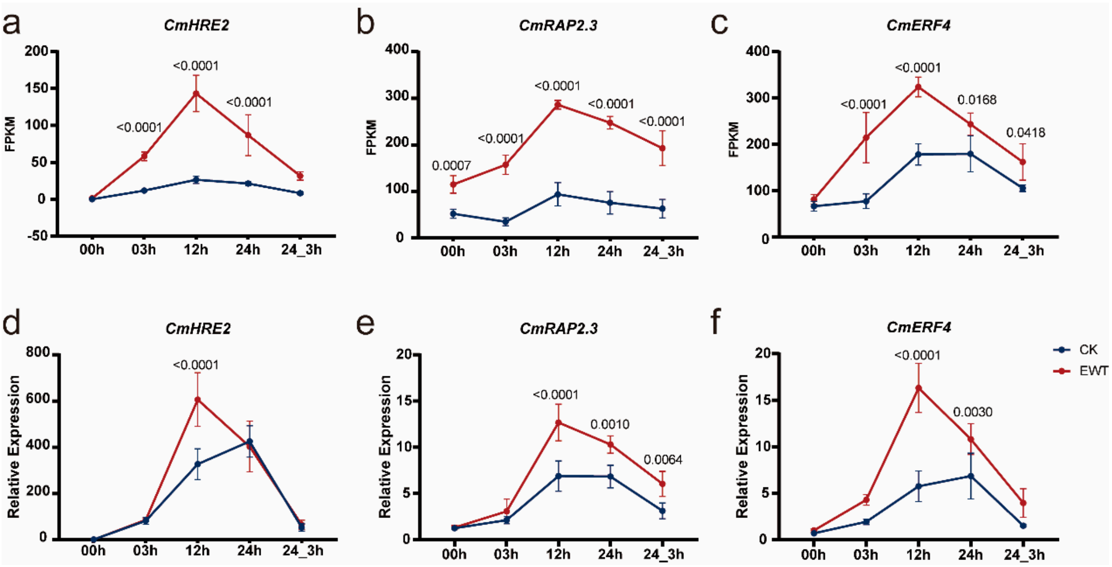

Supplementary Figure S4. Expression of *CmERFs* in roots of CK and EWT after waterlogging.

(a-c) Expression of *CmHRE2* (a), *CmRAP2.3* (b), *CmERF4* (c) in two materials at the indicated time points during waterlogging. Data points show expression levels as average FPKM (Fragments Per Kilobase of exon per Million fragments mapped) in four biological replicates. (d-f) Real-time RT-PCR analysis of relative expression of *CmHRE2* (d), *CmRAP2.3* (e), *CmERF4* (f) in two materials during waterlogging. *CmActin* was served as an internal control. The average values (±s.d., n = 4, biologically independent samples) are shown. Significant differences were determined by two-way ANOVA with Tukey's HSD post hoc analysis. Exact P values are indicated above the bars. CK refers to 'zaoxiaoyangju', EWT refers to a new chrysanthemum variety called 'Enhanced Waterlogging Tolerance'.

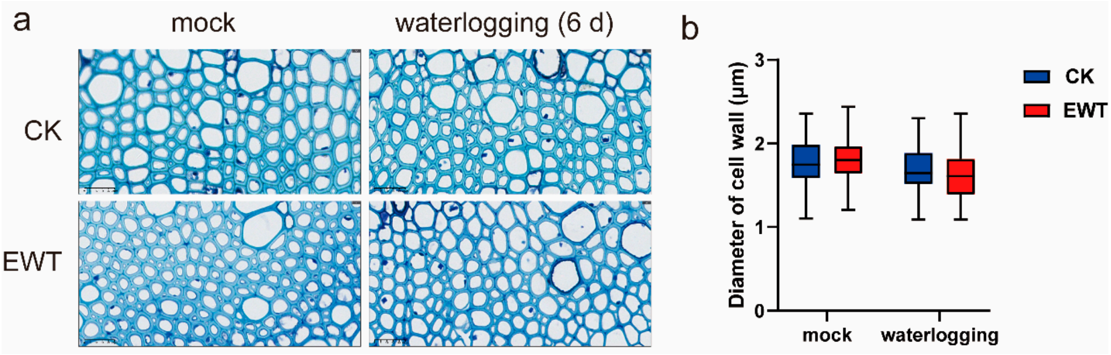

Supplementary Figure S5. Phenotype of vessel cells in EWT and CK before and after waterlogging.

(a) Magnified view of the vessel cells in Fig. 2a. Scale bars, 25 μm. (b) The diameter of vessel cell wall in EWT and CK before and after flooding. Data are displayed as box and whisker plots with individual data points (*n* = 80, biologically independent samples). The error bars represent maximum and minimum values. Center line, median; box limits, 25th and 75th percentiles. Significant differences were determined by two-way ANOVA with Tukey's HSD post hoc analysis. CK refers to 'zaoxiaoyangju', EWT refers to a new chrysanthemum variety called 'Enhanced Waterlogging Tolerance'.
